# Tailgut cyst with adenocarcinoma transition: A rare case report

**DOI:** 10.1097/MD.0000000000020941

**Published:** 2020-07-02

**Authors:** Min Wang, Guoliang Liu, Yu Mu, Hongyu He, Shuang Wang, Jiannan Li

**Affiliations:** a Department of General Surgery; b Operating Theater and Department of Anesthesiology; c Department of Dermatology, The Second Hospital of Jilin University, Changchun, Jilin, China.

**Keywords:** computer tomography, magnetic resonance imaging, pathology, surgery, tailgut cyst

## Abstract

**Rationale:**

Tailgut cyst (TGC) is a rare congenital disease that originates from residues of the tail intestine during the embryonic period. Most TGCs are benign lesions and the malignant transition is very rare.

**Patient concerns:**

A 50-year-old woman attended our department complaining of defecation difficulty for more than 2 months. She reported irregular defecation with a small amount of liquid stool, 3 to 4 times per day.

**Diagnosis:**

Biochemical analysis showed high levels of carcinoembryonic antigen (79.89 ng/mL; normal, 0–3 ng/mL) and carbohydrate antigen 199 (57.60 U/mL; normal, 0–35 U/mL). Abdominal computer tomography and magnetic resonance imaging showed a large cystic mass with enhanced signals. Post-surgical histopathology indicated that the mass was a TGC with adenocarcinoma transition.

**Interventions:**

The cyst was completely resected. Symptomatic treatment was further performed, and the patient recovered well.

**Lessons:**

We reported a rare case of a large TGC with adenocarcinoma transition. CT, MRI, and histopathology are important to diagnose TGC. Complete surgical resection is the first choice to treat TGC.

## 1 Introduction

Tailgut cyst (TGC), also known as retrorectal cystic hamartoma, is a rare congenital disease that originates from residues of the tail intestine during the embryonic period.^[1]^ TGC is common in adult women and the ratio of female to male patients is 3:1 to 4:1.^[2]^ TGCs are often located in the space behind the rectum and ahead of the sacrum. In addition, the upper boundary of the space is the peritoneal reflex; the lower boundary is the levator ani muscle; and the bilateral boundaries are the bilateral ureters, iliac vessels, and sacral nerve roots.^[3,4]^ TGC was first reported by Middelporf in 1885 and was considered as a remnant of the tail intestine.^[5]^ Most TGCs are benign lesions and malignant transition is very rare.^[6]^ In this study, we report a rare case of a large TGC with adenocarcinoma transition. We summarize its clinical features, diagnosis, and treatment methods.

## 2 Case report

This study was approved by the Ethics Committee and institutional Review Board of the Second Hospital of Jilin University, Changchun, China. The patient provided informed consent for publication of the case.

A 50-year-old woman attended our department with defecation difficulty for more than 2 months. She complained about irregular defecation with a small amount of liquid stool, 3 to 4 times per day. There was no blood or mucus in the stool. One month earlier, the symptoms of irregular defecation worsened, and abdominal distention occurred that was mainly located in the middle and lower abdomen. The abdominal distention was slightly alleviated after defecation. In addition, there was no accompanying abdominal pain. The patient denied any history of hypertension, coronary heart disease, diabetes, or infectious diseases. She had received a Cesarean section 25 years ago and received surgical treatment for teratoma 12 years ago. A digital rectum examination (lithotomy position) indicated a 3 × 3 cm sized mass that showed good mobility upon touch, which was at the 6 o’clock orientation and was 4 cm from the anal edge. The upper margin of the tumor was not tough.

**Figure 1:**
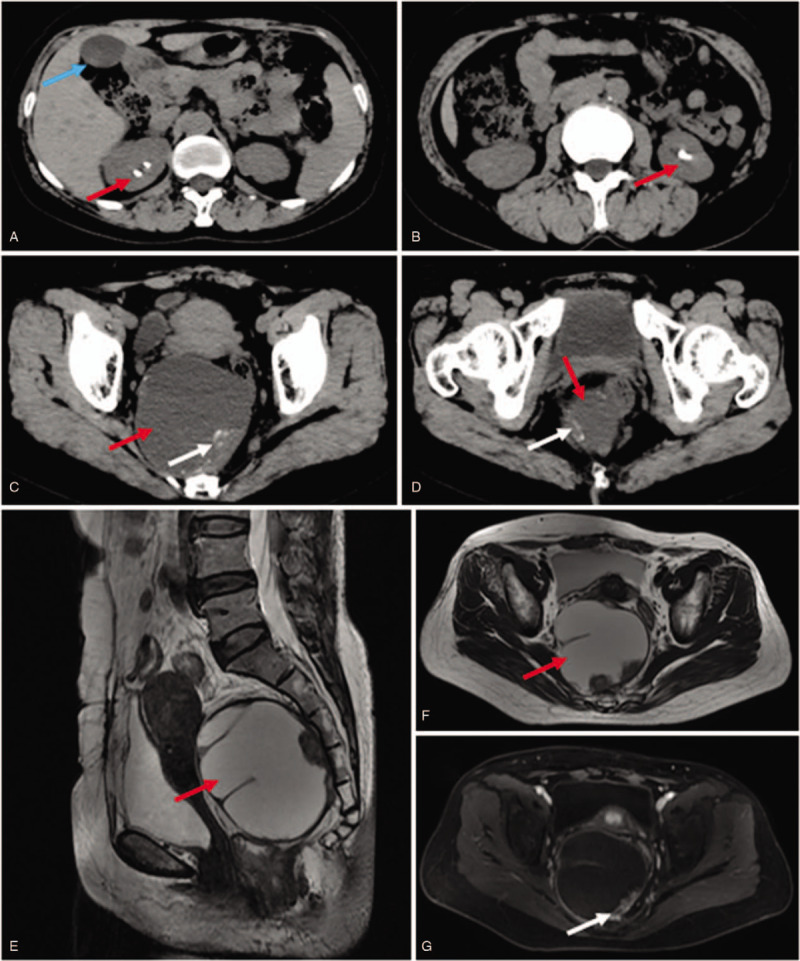
Imaging examination. (A) CT showing gallstones (blue arrow) and right kidney stones (red arrow). (B) The left kidney stone (red arrow). (C) CT showing a 80 × 80 mm sized mass with low density shadows in the pelvic cavity (red arrow). The white arrow indicates the high-density signals within the lesion. (D) The uterus and rectum were compressed. (E) MRI showing a large cystic shadow (red arrow) with long T1 and T2 signals. (F) The boundary between the lesion and the right accessory was unclear. A red arrow indicates the lesion. (G) The separation wall and the nodules (white arrow) of the lesion were significantly enhanced. CI = computed tomography, MRI = magnetic resonance imaging.

The patient's carcinoembryonic antigen (CEA) level was 79.89 ng/mL (normal, 0–3 ng/mL) and her carbohydrate antigen 199 level was 57.60 U/mL (normal, 0–35 U/mL). There were no other abnormal biochemical indicators. A pelvic ultrasound examination revealed a 90 × 80 mm sized echoic mass in the pelvic cavity that had many separations. Abdominal computed tomography (CT) found gallstones (Fig. 1A), bilateral kidney stones (Fig. 1A and B), and a 80 × 80 mm sized mass with low-density shadows in the pelvic cavity (Fig. 1C). High density shadows with stripes were also observed. Furthermore, the uterus and rectum were compressed (Fig. 1D). Magnetic resonance imaging (MRI) showed a large cystic shadow with long T1 and T2 signals (Fig. 1E). The size of the tumor was 83 × 88 × 85 mm and the boundary between the lesion and the right accessory was unclear (Fig. 1F). An enhanced MRI scan showed that the separation wall and the nodules of the lesion were significantly enhanced (Fig. 1G). An electric coloscopy revealed chronic colitis and colorectal polyps. Based on the symptoms and clinical examination, our clinical team considered the lesion to be a presacral cyst.

**Figure 2:**
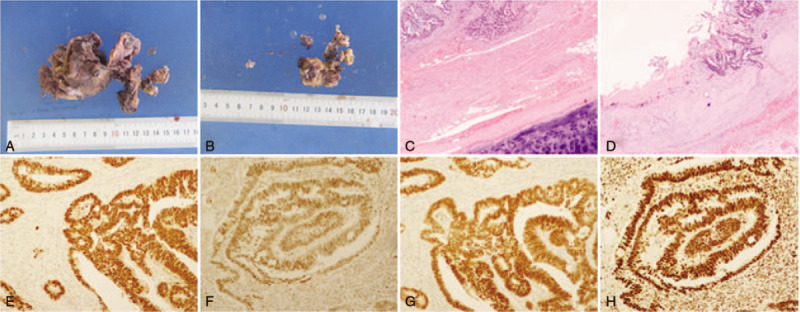
Pathological examination. (A) and (B) show the resected TGC. (C) and (D) show that the lesion was a TGC accompanied by moderately differentiated adenocarcinoma. (E and F) Positive staining for CK20 (E), CK7 (2F), CDX2 (G), and Ki67 (H). CDX2 = caudal type homeobox 2, CK7 = cytokeratin 7, CK20 = cytokeratin 20, TGC = tailgut cyst.

This patient underwent surgical resection of the lesion. General anesthesia was performed with the patient in the folding knife position. The presacral tumor was resected via the transsacrococcygeal approach. An incision of approximately 8 cm was made posterior sagittal and located in the midline. The skin, subcutaneous tissue, and fascia were cut. The tip of the tailbone was cut off to expose the space, which was anterior to the sacrum and posterior to the rectum. In the space, an irregular cystic mass with no internal pus was found. The mass was completely and carefully resected (Fig. 2A and B) with no injury to the nearby rectum and uterus. A drainage tube was placed in the space anterior to the sacrum.

Histopathology indicated that the lesion was a TGC accompanied by moderately differentiated adenocarcinoma (Fig. 2 C and D). Immunohistopathological showed positive staining for cytokeratin 20 (Fig. 2E), cytokeratin 7 (Fig. 2F), caudal type homeobox 2 (Fig. 2G), and marker of proliferation Ki-67 (MKI67/Ki67 (Fig. 2H). The patient recovered well and was discharged at 7 days post-surgery. The patient received post-surgical adjuvant radiotherapy. To date, the follow-up time has been approximately 6 months, and no local recurrence or distant metastasis has been found.

## 3 Discussion

TGC is a residue of the primitive tail intestine and is a rare congenital disease. The etiology of TGC is not clear. Hjermstad et al believed that the incomplete degeneration of tail intestine or neural tube might lead to the formation of a TGC.^[5]^

TGCs have various clinical manifestations, none of which are specific.^[7,8]^ Approximately half of patients with a TGC complained about abdominal mass and pain; however, the others did not have any symptoms.^[1,5]^. The size and location of the cyst, whether the surrounding tissues and organs are involved, and whether the cyst is associated with infection and malignancy, play an important role in the symptoms of TGC.^[9]^ Compression of the rectum by TGC can lead to defecation difficulty, pain in the lower abdomen, and constipation.^[10]^ In our case, the rectum was seriously compressed, and the patient complained about defecation difficulty. Occasionally, large TGCs can cause obstruction of the pelvic outlet, thus leading to dystocia or sciatica.^[11]^ Other patients also complained about abdominal or vaginal pain.^[12]^

Pelvic ultrasound examination, CT, and MRI are the main examination methods used to diagnose a TGC. Abdominal CT and MRI are valuable to understand the nature of the mass, its relationship with the surrounding tissues, and to determine whether the cysts are undergoing carcinoma transition.^[13,14]^ MRI is better than CT in locating the cyst, determining the nature of the contents of the cyst, and identifying the relationship between the cyst and the surrounding tissues.^[13]^ Typical MRI findings include long T1 and T2 signals and multilocular manifestations.^[15]^ High levels of CEA might indicate the carcinoma transition of a TGC. Postsurgical histopathology remains the “gold standard” to diagnose TGC. In our study, MRI showed a large and multilocular mass with long T1 and T2 signals. The CEA level was much higher than normal. The histopathological examination confirmed the adenocarcinoma transition of the TGC.

Surgical management is typically required for nearly all presacral tumors. Complete removal of the epithelial lining and a clear margin of excision are vital for TGC resection. Adequate preoperative preparation can effectively reduce the risk of perioperative surgery and is the basis for successful surgery. When a TGC is close to the rectum, it is easy to damage the rectum during surgery. Therefore, bowel preparation before surgery is important. When the mass is located deep and close to the anterior sacral venous plexus and internal iliac vessels, major bleeding is the greatest risk of surgery. Therefore, blood preparation before surgery is also vital. Ureteral catheterization is also required if there is a close relationship between the TGC and the ureter. Complete resection of the tumor is most important to treat TGC. The experiences of TGC resection are summarized below.

(1) Good exposure of surgical field and sufficient operating space are vital for successful surgery. Based on the anatomical site of the TGCs, there are 4 main surgical approaches, including the sacrococcygeal approach, the single abdominal approach, the combined sacrococcygeal approach, and the anal approach.^[16]^
(2) The TGCs should be sharply separated from the outside to inside along the TGC envelope.
(3) For cysts that are large, deep, and difficult to remove, punctuation to reduce the cyst volume should be performed.
(4) If the cysts have infiltrated nearby organs or tissues, combined organ resection can be performed.
(5) The principle of surgery is to completely remove the cyst; however, for patients whose cysts cannot be completely removed, electrocoagulation cauterization can be used.

TGCs with malignant transformation are always diagnosed using postsurgical histopathology. The 3 most common types of malignant transformations in TGCs are neuroendocrine tumor, adenocarcinoma, and squamous carcinoma.^[16]^ For TGCs with malignant transformation, post-surgical adjuvant therapy is suggested to kill the residual tumor tissues and improve patient prognosis.^[16]^ For TGCs with neuroendocrine tumor transformation, the administration of postoperative somatostatin might improve patient prognosis.^[17]^ In addition, tyrosine kinase inhibitors and an oral mechanistic target of rapamycin inhibitors have been found to increase patients’ progression-free survival of patients.^[18]^ For TGCs with adenocarcinoma and squamous carcinoma transformation, surgical resection followed by radiotherapy and chemotherapy is still the main treatment method.^[16]^ However, few cases of TGCs with malignant transformation are reported; therefore, the metastatic potential of TGCs with malignant transformation is unclear.

In the present study, we reported a rare case of a large TGC with adenocarcinoma transition. CT, MRI, and histopathology confirmed the diagnosis of TGC. Complete surgical resection of the TGC was performed successfully. We summarized the clinical manifestations, diagnostic methods, and treatment experiences of TGC.

## Author contributions

Jiannan Li and Shuang Wang designed this study. Min Wang wrote this study. Guoliang Liu, Yu Mu, Hongyu He, and Min Wang performed the surgery. Jiannan Li confirmed the final form of this study.
